# A Comparative Assessment Between High-resolution Ultrasonography and Field Magnetic Resonance Imaging in Supraspinatus Tear Cases and Its Arthroscopic Correlation

**DOI:** 10.7759/cureus.5627

**Published:** 2019-09-11

**Authors:** Tushar Sabharwal, Sachin Khanduri, Shahla Khan, Mushahid Husain, Anchal Singh, Ahmad Umar Khan, Syed Zain Abbas, Harshika Singh

**Affiliations:** 1 Radiodiagnosis, Era's Lucknow Medical College and Hospital, Lucknow, IND; 2 Radiology, Era's Lucknow Medical College and Hospital, Lucknow, IND

**Keywords:** supraspinatus tear, magnetic resonance imaging, ultrasonography, full thickness tear, partial thickness tear, partial thickness tear

## Abstract

Background

Diagnosis of a supraspinatus tear in patients presenting with shoulder pain is a difficult task and often requires the help of an MRI. However, in recent years, high-resolution ultrasonography (USG) has been utilized as a cheaper yet sensitive alternative. The aim of the study is to provide a comparative assessment of supraspinatus tears between USG and MRI in relation to arthroscopic results.

Methods

A total of 60 patients with shoulder pain for the last three months or more scheduled to undergo arthroscopic surgery for their shoulder disorder were enrolled; those having any congenital deformity of the shoulder or having any contradiction to an MRI were excluded from the assessment. All the patients underwent high-resolution ultrasonography (HRUSG) and MRI evaluation. Both the USG and MRI findings were correlated with the arthroscopic findings.

Results

On ultrasonography, 34 (56.67%) full-thickness tears and 22 (36.67%) partial-thickness tears of the supraspinatus were detected. On MRI, 36 (60.0%) were diagnosed as a full-thickness tear and 20 (33.33%) as a partial-thickness tear. After arthroscopy, 36 (60.00%) were confirmed as a full-thickness tear and 20 (33.33%) as a partial-thickness tear of the supraspinatus. For a full-thickness tear, the sensitivity and specificity of USG and MRI were 95.0% and 92.5%, and 85% and 92.5%, respectively. For a full-thickness tear, the sensitivity and specificity of the modalities were 94.4% and 100%, respectively.

Conclusion

HRUSG and MRI both had high comparable accuracy for detection of a supraspinatus tear, however, HRUSG had an edge over MRI in the detection of a partial tear.

## Introduction

Shoulder pain is a commonly reported concern. In the general population, as many as 20% of adults experience shoulder pain symptoms at a given time, and many of those adults do not consult a doctor [1]. The expected incidence rate is 14.7 per 1,000 patients per year. The most common causes of shoulder pain in primary care are reported to be rotator cuff disorders [2]. The rotator cuff muscles include the supraspinatus, infraspinatus, teres minor, and subscapularis, and function dynamically to stabilize the shoulder joint. The supraspinatus muscle carries out the initial 15 to 30 degrees of abduction, the infraspinatus performs external rotation, and the teres minor and subscapularis handle internal rotation of the shoulder.

Ultrasonography (USG) is an effective tool for evaluation of rotator cuff pathologies. Secondary USG signs of a full-thickness supraspinatus tear include a cortical irregularity of greater tuberosity and joint effusion. A partial-thickness supraspinatus tear is characterized by tendon non-visualization, greater tuberosity cortical irregularity, and cartilage interface signs. The sensitivity of different USG signs for prediction of full-thickness tears versus partial-thickness tears or no tear is highly variable, ranging from as low as 0% (intrasubstance) be 86% (cortical irregularity) [3]. Multidetector computed tomography (MDCT), an improvement over conventional single-slice computed tomography, is useful in carrying out noninvasive techniques like arthroscopy [4]. MRI of the shoulder is highly sensitive and specific for the detection of full-thickness and partial-thickness supraspinatus tendon tears [5]. Based on this, the present study carried out a comparative assessment of supraspinatus tears between USG and MRI in relation to arthroscopic results.

## Materials and methods

The present study was carried out at the Department of Radiology, Era’s Lucknow Medical College and Hospital (ELMCH), in the socioeconomic underprivileged suburban and rural population of Lucknow. Patients of either gender of ages 18 to 60 years who visited with concerns of shoulder pain lasting greater than three months were selected for the study. Patients with a history of any congenital deformity of the shoulder were excluded. The project was approved by the Institutional Ethical Committee of ELMCH (#ELMC/EC/RCell/2014/220). Informed consent was obtained from all the participants. USG and MRI evaluations were done for all patients by two separate independent experts.

USG was performed using a GE Voluson P8 (GE Healthcare, Little Chalfont, United Kingdom) ultrasonography machine with a high frequency 7-12 MHz linear electronic array transducer. The examination method, as described by Jacobson, was followed [6]. The arm was examined abducted in a sitting position, and the probe was applied anteriorly, posteriorly, and superiorly.

On USG, full-thickness tears appear as hypoechoic/anechoic defects in the tendon. Due to fluid replacing the tendon, the cartilage shadow is accentuated, giving a double cortex or cartilage interface sign. Also, due to the defect, overlying peribursal fat dips down into the tendon gap, creating a sagging peribursal fat sign [7].

On MRI, complete tears appear as hyperintense signal area within the tendon on T2 weighted (T2W), fat suppressed, and gradient echo (GRE) sequences. Complete tears extend from the articular to the bursal surface, most commonly in the supraspinatus tendon. The presence of a tendon defect filled with fluid is the most direct sign of rotator cuff tear.

In a complete tear, there is no communication between the subacromial-subdeltoid (SASD) bursa and the glenohumeral joint. They can be either partial or intratendinous. In an intratendinous tear, the split is only within the tendon itself, and there is no communication with the SASD bursa or the shoulder joint. In partial tears, some tendinous fibers on the articular or bursal surface are interrupted. Indirect signs on MRI include subdeltoid bursal effusion, medial abduction of biceps, fluid along the biceps tendon, and diffuse loss of the peribursal fat plane.

After USG and MRI, arthroscopy was performed to confirm the results as it is considered the gold standard. Data analysis was done using IBM SPSS Statistics for Windows, Version 20.0 (IBM Corp., Armonk, NY). Diagnostic efficacy was calculated in terms of sensitivity, specificity, positive predictive, negative predictive, and accuracy values.

## Results

A total of 60 patients aged 25 to 60 years were enrolled in the study. The mean age of the patients was 45.37 ± 8.70 years. The majority of patients were men (68.33%). The mean duration of symptoms was 9.28 ± 4.31 months (range, from three to 24 months). The majority had involvement of the right side (n=46; 76.67%). The patient’s dominant side was involved in 47 (78.33%) cases. A total of 11 (18.33%) cases had a history of diabetes. There were 32 (53.33%) patients with a history of trauma. Tenderness was reported as the presenting concern by 21 (35%) cases. The majority (63.33%) reported night pain as the presenting concern. A total of 27 (45%) had a full range of movement. Among others, nine (15%) had a range of movement of either greater than 45 degrees or 30-45 degrees, respectively, and 15 (25%) had a range of movement less than 30 degrees (Table 1).

**Table 1:** Demographic profile and general characteristics of patients SN - serial number; SD - standard deviation

SN	Characteristic	Statistic
1	Mean age ± SD (range) in years	45.37±8.70 (25-60)
2	Men : women	38 (68.33%) : 22 (36.67%)
3	Mean duration of symptoms ± SD (range) in months	9.28±4.31 (3-24)
4	Side involved (left, right)	14 (23.33%), 46 (76.67%)
5	Dominant side involvement	47 (78.33%)
6	Medical history (diabetes, trauma)	11 (18.33%), 32 (53.33%)
7	Presenting signs and symptoms (tenderness, night pain)	21 (35.00%), 38 (63.33%)
8	Range of movement (full, < 30°, 30-45°, > 45°)	27 (45.00%), 15 (25.00%), 9 (15.00%), 9 (15.00%)

On USG, the tendon could not be visualized in 32 (53.33%) cases. In 23 (38.33%), abnormal echogenicity was seen. Irregular margins were seen in 25 (41.67%) cases and thinning in 29 (48.33%) cases. A total of 27 (45%) had cortical irregularity while 13 (21.7%) had a cartilage interface sign. Joint fluid and bursal fluid could be visualized in 49 (81.67%) and 36 (60%) cases, respectively. Acromioclavicular joint (ACJ) hypertrophy was seen in four (6.67%) and impingement in five (8.33%) cases. The location of the tear could not be located in four (6.67%) cases. In the majority of cases, (53.33%) a total tear was seen; bursal, intrasubstance and focal tear could be located in seven (11.67%), five (8.33%), and two (3.33%) cases, respectively. Based on characterization and localization of USG, a total of 34 (56.67%) were diagnosed as a full-thickness tear of the supraspinatus while 22 (36.67%) were diagnosed as a partial-thickness tear of the supraspinatus. A total of three (5%) cases were diagnosed as tendinosis of the supraspinatus, and one (1.67%) case was diagnosed as calcified tendinitis (Table 2).

**Table 2:** Ultrasonographic characteristics, location of tear and USG diagnosis SN - serial number; ACJ - acromioclavicular joint; USG - ultrasonography

SN	Characteristics / Location	Number of patients	Percentage
A	Characteristics		
1	Tendon non-visualization	32	53.33
2	Abnormal echogenicity	23	38.33
3	Irregular margins	25	41.67
4	Thinning	29	48.33
5	Cortical irregularity	27	45.00
6	Cartilage interface sign	13	21.70
7	Joint fluid	49	81.67
8	Bursal fluid	36	60.00
9	ACJ hypertrophy	4	6.67
10	Impingement	5	8.33
B	Location		
1	Not detected	4	6.67
2	Articular	10	16.67
3	Bursal	7	11.67
4	Intrasubstance	5	8.33
5	Focal	2	3.33
6	Total tear	32	53.33
C	USG diagnosis		
1	Calcified tendinitis	1	1.67
2	Full-thickness tear of supraspinatus	34	56.67
3	Partial-thickness tear of supraspinatus	22	36.67
4	Tendinosis of supraspinatus	3	5.00

On MRI, joint effusion was seen in 51 (81%) cases. A total of 45 (75%) had irregular borders, 38 (63.33%) each showed discontinuity, reduced thickness, and reduced subacromial space, respectively. There were 35 (58.33%) showing ACJ hypertrophy, and 28 (46.67%) showed SASD fluid. A total of 12 (20%) had muscle atrophy, 11 (18.33%) had labral bursal and three (5%) showed involvement of the axillary lymph node. MRI could not localize the tear in three (5%) cases. A total tear was seen in 34 (56.67%) cases while the tear was articular in 10 (16.67%), bursal in seven (11.67%), intrasubstance visualized in four (6.67%), and focal in two (3.33%) cases. Correspondingly, the MRI diagnosis was full-thickness tear in 36 (60%), partial-thickness tear in 20 (33.33%) and tendinosis in four (6.67%) cases (Table 3).

**Table 3:** MRI characteristics, location of tear and MRI diagnosis SN - serial number; SASD - subacromial-subdeltoid; ACJ - acromioclavicular joint; USG - ultrasonography

SN	Characteristics / Location	Number of patients	Percentage
A	Characteristics		
1	Discontinuity	38	63.33
2	Irregular border	45	75.00
3	Reduced thickness	38	63.33
4	Muscle atrophy	12	20.00
5	Reduced subacromial space	38	63.33
6	Joint effusion	51	81.00
7	SASD fluid	28	46.67
8	ACJ hypertrophy	35	58.33
9	Labral bursal	11	18.33
10	Axillary lymph node	3	5.00
B	Location		
1	Not detected	3	5.00
2	Articular	10	16.67
3	Bursal	7	11.67
4	Intrasubstance	4	6.67
5	Focal	2	3.33
6	Total tear	34	56.67
C	MRI diagnosis		
1	Calcified tendinitis	0	0.00
2	Full-thickness tear of supraspinatus	36	60.00
3	Partial-thickness tear of supraspinatus	20	33.33
4	Tendinosis of supraspinatus	4	6.67

Arthroscopically, 36 (60%) cases were diagnosed as full-thickness tears, 20 (33.33%) as partial-thickness tears, three (5%) as tendinosis, and one (1.67%) as calcified tendinitis (Table 4).

**Table 4:** Arthroscopic diagnosis SN - serial number

SN	Diagnosis	Number of patients	Percentage
1	Calcified tendinitis	1	1.67
2	Full thickness tear of supraspinatus	36	60.00
3	Partial-thickness tear of supraspinatus	20	33.33
4	Tendinosis of supraspinatus	3	5.00

On evaluating the diagnostic efficacy of ultrasonography, it was found to have 100% sensitivity, 100% specificity, 100% positive predictive value (PPV), 100% negative predictive value (NPV), and 100% diagnostic accuracy for the diagnosis of calcified tendinitis. The sensitivity, specificity, PPV, NPV and diagnostic accuracy for diagnosis of a full-thickness tear of the supraspinatus was 94.4%, 100.0%, 100%, 92.3%, and 96.7%, respectively. For the diagnosis of a partial-thickness tear of the supraspinatus, these values were 95.0%, 92.5%, 86.4%, 97.4%, and 93.3%. For the diagnosis of tendinosis of the supraspinatus, the values were 66.7%, 98.2%, 66.7, 98.2%, and 96.7%, respectively. On the other hand, MRI was found to have 0% sensitivity, 100% specificity, 98.3% NPV, and 98.3% diagnostic accuracy for the diagnosis of calcified tendinitis. The sensitivity, specificity, PPV, NPV, and diagnostic accuracy for the diagnosis of a full-thickness tear of the supraspinatus was 94.4%, 100.0%, 100%, 92.3%, and 96.7%, respectively, while for the diagnosis of a partial-thickness tear of the supraspinatus, these values were 85.0%, 92.5%, 85.0% 92.5%, and 90.0%, respectively, and for the diagnosis of tendinosis of the supraspinatus, they were 100.0%, 98.2%, 75.0, 100.0%, and 98.3%, respectively (Table 5).

**Table 5:** Diagnostic efficacy of USG and MRI against arthroscopy USG - ultrasonography; PPV - positive predictive value; NPV - negative predictive value

Diagnosis	True +ve	False –ve	False +ve	True –ve	Sensitivity	Specificity	PPV	NPV	Diagnostic accuracy
USG									
Calcified tendinitis	1	0	0	59	100.0	100.0	100.0	100.0	100.0
Full-thickness tear of supra-spinatus	34	2	0	24	94.4	100.0	100.0	92.3	96.7
Partial-thickness tear of supraspinatus	19	1	3	37	95.0	92.5	86.4	97.4	93.3
Tendinosis of supraspinatus	2	1	1	56	66.7	98.2	66.7	98.2	96.7
MRI									
Calcified tendinitis	0	1	0	59	0.0	100.0	–	98.3	98.3
Full-thickness tear of supraspinatus	34	2	0	24	94.4	100.0	100.0	92.3	96.7
Partial-thickness tear of supraspinatus	17	3	3	37	85.0	92.5	85.0	92.5	90.0
Tendinosis of supraspinatus	3	0	1	56	100.0	98.2	75.0	100.0	98.3

## Discussion

A shoulder injury is a generalized term with a number of underlying pathologies. Supraspinatus injury is the most common cause of shoulder pain. However, clinical evaluation alone is insufficient to provide useful information about the extent, type, and nature of supraspinatus injury [8].

Signs like cortical irregularity and joint fluid are helpful in the diagnosis of full-thickness supraspinatus tears while partial-thickness supraspinatus tears are characterized by tendon non-visualization, cortical irregularity, and cartilage interface sign. This demonstrates that independent signs are not confirmatory and require a skillful understanding of a combination of factors to establish an accurate diagnosis.

The location and span of each tear were assessed through USG. A total of 32 (53.33%) cases showed the span of tears to be over the complete supraspinatus as indicated by the USG characteristics. A total of 34 (56.67%) patients were diagnosed with a full-thickness shoulder injury.

Given the reemergence of USG as a diagnostic modality for the evaluation of rotator cuff injury, the present study was carried out as a comparative assessment of supraspinatus tears with USG and MRI and correlated with arthroscopic results as the gold standard.

In the present study, the age of the patients ranged from 25 to 60 years, and the mean age was 45.37 years. The upper age in the present study was limited based on the inclusion criteria. Rotator cuff tears have been reported to affect almost all age groups; in various studies, the mean age has been reported to be above 50 years. However, it has been most commonly observed in middle-aged individuals.

As seen in previous studies, the condition is more common in men vs. women. Gender ratios have been reported (men : women) ranging from 1.31 to 2.1. Our study found a gender ratio of 1.73. The duration of symptoms ranged from two to 24 months, with a mean duration of 9.28 ± 4.31 months. The majority of patients had a duration of symptoms in the seven to 12 months range. In clinical practice, MRI is still considered to be the mainstay of rotator cuff injury diagnosis and, owing to its high cost, patients generally wait until alternative remedies fail to provide relief.

The right shoulder was affected more (76.77%) than the left shoulder (23.33%). The high incidence of the right shoulder as the affected side could be primarily attributed to the prevalence of right-handed patients (98.3%). Clinical features like tenderness (35%) and night pain (65.33%) were reported. Features like night pain are suggestive of the initial phase of adhesive capsulitis [2].

The most common USG findings were bursal fluid (60%), followed by tendon non-visualization (53.33%), joint fluid (53.33%), thinning (48.33%), cortical irregularity (45%), irregular margins (41.67%), abnormal echogenicity (38.33%), and cartilage interface sign (21.7%), respectively. Signs like cortical irregularity and joint fluid can be indicative of a full-thickness tear. In a total of four (6.67%) cases, USG did not detect any tear at all. The articular (16.67%) and bursal (11.67%) regions were a common location. The focal and intrasubstance location was involved in two (3.33%) and five (8.33%) cases, respectively. On MRI, the major findings were irregular borders (75%), reduced subacromial space (63.33%), reduced thickness (63.33%), discontinuity (63.33%), ACJ hypertrophy (58.33%), SASD fluid (46.47%), joint effusion (45%), muscle atrophy (20%), labral bursa (18.33%), and axillary lymph node (5%), respectively.

**Figure 1:**
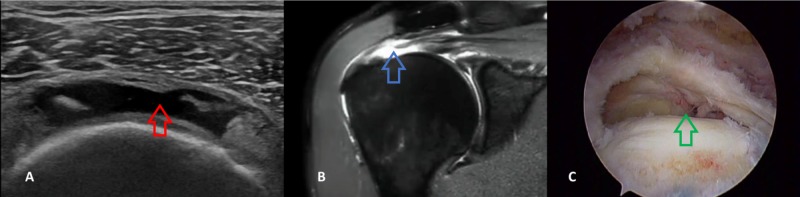
A 52-year-old male patient with a full-thickness (total) supraspinatus tear Longitudinal HRUSG image of the supraspinatus tendon reveals a hypoechoic discontinuity of the supraspinatus tendon (A: red arrow) extending from the articular to bursal surface. Note the cortical irregularity. STIR coronal MRI image of the shoulder joint reveals a full-thickness tear in the supraspinatus muscle with joint effusion (B: blue arrow). The arthroscopic image demonstrates a total full-thickness supraspinatus tear (C: green arrow). HRUSG - high-resolution ultrasonography; STIR - short TI Inversion Recovery

**Figure 2:**
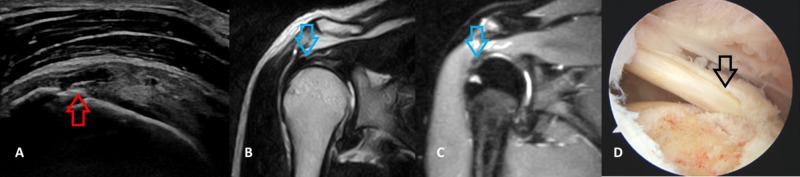
A 36-year-old male patient with a bursal partial-thickness supraspinatus tear A: longitudinal HRUSG image of the supraspinatus tendon reveals a hypoechoic discontinuity of the supraspinatus tendon extending on the bursal surface and extending up to the greater tuberosity and not to the articular surface (red arrow). Note the cortical irregularity. B and C: T2 & STIR coronal MRI image of the shoulder joint reveals a partial-thickness tear in the supraspinatus muscle (blue arrow). The arthroscopic image demonstrates atotalbursal partial-thickness supraspinatus tear (black arrow). HRUSG - high-resolution ultrasonography; STIR - short TI Inversion Recovery

**Figure 3:**
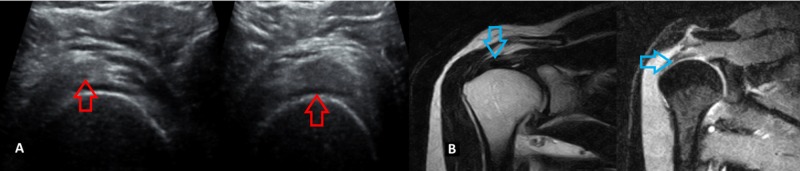
A 40-year-old male patient with supraspinatus tendinosis Longitudinal HRUSG image of the supraspinatus tendon reveals a bulky and heterogeneous tendon with joint effusion (A: red arrow). T2 and STIR coronal MRI image of the shoulder joint reveals an increased thickness with increased signal intensity on coronal T2 and STIR images (B: blue arrow). HRUSG - high-resolution ultrasonography; STIR - short TI Inversion Recovery

The most specific sign of a full-thickness rotator cuff tear is the visualization of a complete defect in the tendon, extending from the articular surface, completely through to the bursal surface (Figure 1) [8, 9]. Secondary signs of full-thickness rotator cuff tears include fluid in the SASD bursa and muscle atrophy. On tears of the supraspinatus, 22 (3667%) were diagnosed as partial-thickness tears (Figure 2). Three (5%) were diagnosed as tendinosis of the supraspinatus (Figure 3) and one (1.67%) was diagnosed as calcific tendinosis.

 

 

MRI diagnosis was similar to that of USG, however on MRI calcific tendinosis could not be detected in any case while full-thickness tears were detected in 36 (60%) and partial-thickness tears in 10 (33.33%) cases. A total of four (6.67%) cases were diagnosed as tendinosis. One of the reasons for the inability of MRI to diagnose calcific tendinosis lies in the fact that calcification appears as hypointense or heterogeneous areas and can therefore not be reliably distinguished from signal artifacts resulting from tissue interfaces or hemorrhage.

Finally, on arthroscopy, a total of 36 (60%) cases were found to be full-thickness tears, 20 (33.33%) partial-thickness tears, three (5%) tendinosis, and one (1.67%) calcific tendinosis.

On correlating the USG finding with arthroscopy, an agreement was seen in 56 out of 60 (93.33%) cases. USG missed two cases of full-thickness tear and interpreted them as partial-thickness tears. One case of a partial-thickness tear was interpreted as tendinosis, and one case of tendinosis was interpreted as a partial-thickness tear.

In the present study, despite certain limitations, both USG and MRI showed comparable diagnostic efficiency for full-thickness and partial-thickness tears. For full-thickness tears, both USG and MRI had a sensitivity and specificity of 94.5% and 100%. For partial-thickness tears, USG had a sensitivity and specificity of 95% and 92.5% while MRI had a sensitivity and specificity of 85% and 92.5%, respectively. Therefore, in the present study, we found that USG had an edge over MRI in the detection of a partial-thickness tear. However, a systematic review by Dinnes et al. including 38 cohort studies on USG showed that USG is the most accurate when used for the detection of full-thickness tears. However, the sensitivity was lower for the detection of partial-thickness tears [10].

Before this study, few facilities around the world viewed on USG as a useful and reliable modality for the diagnosis of a supraspinatus tear. In subsequent years, more evidence had started to come up supporting the view that USG either has similar or even better diagnostic efficacy for detection of full and partial tears of the supraspinatus. Taken together, these data demonstrate that shoulder ultrasound, in the hands of an experienced radiologist with modern high-resolution equipment, is highly sensitive in differentiating complete tears and partial-thickness tears [11].

Being a dynamic technique, USG provides useful information to an expert and skilled radiologist, who can transform it into the correct diagnosis. Thus USG, despite being useful and equally comparable for tear diagnosis and relatively better for the diagnosis of calcific tendinosis, underlines the need for adequately trained workforce to handle it. The fact that the present study was carried out at a tertiary care institution under the supervision of a radiologist having more than 25 years of experience, highlights the importance of operator experience. Several previous researchers have also emphasized that a skilled operator can produce comparable results between MRI and USG [12]. The present study supports the contemporary evidence that USG is comparable if not better than MRI and can be recommended as the first-line diagnostic modality in cases of shoulder pain.

## Conclusions

HRUSG and MRI, both had high comparable accuracy for detection of supraspinatus tear, however, HRUSG had an edge over MRI in the detection of a partial tear. 

## References

[1] Pope DP, Croft PR, Pritchard CM, Silman AJ (1997). Prevalence of shoulder pain in the community: the influence of case definition. Ann Rheum Dis.

[2] Mitchell C, Adebajo A, Hay E, Carr A (2005). Shoulder pain: diagnosis and management in primary care. BMJ.

[3] Jacobson JA, Lancaster S, Prasad A, van Holsbeeck MT, Craig JG, Kolowich P (2004). Full-thickness and partial-thickness supraspinatus tendon tears: value of US signs in diagnosis. Radiology.

[4] Fritz J, Fishman E, Fayad L (2014). MDCT arthrography of the shoulder. Semin Musculoskelet Radiol.

[5] Magee T, Williams D (2006). 3.0-T MRI of the supraspinatus tendon. Am J Roentgenol.

[6] Jacobson JA (2011). Shoulder US: anatomy, technique, and scanning pitfalls. Radiology.

[7] MoosikasuwanJB MoosikasuwanJB, Miller TT, Burke BJ (2005). Rotator cuff tears: clinical, radiographic, and US findings. Radio Graphics.

[8] Matthews TJW, Hand GC, Rees JL, Athanasou NA, Carr AJ (2006). Pathology of the torn rotator cuff tendon. J Bone Jt Surg.

[9] Farley TE, Neumann CH, Steinbach LS, Jahnke AJ, Petersen SS (1992). Full- thickness tears of the rotator cuff of the shoulder: diagnosis with MR imaging. AJR Am J Roentgenol.

[10] Dinnes J, Loveman E, McIntyre L, Waugh N (2003). The effectiveness of diagnostic tests for the assessment of shoulder pain due to soft tissue disorders: a systematic review. In NIHR Health Technology Assessment Programme: Executive Summaries.

[11] Sanders TG, TirmanPFJ TirmanPFJ, Feller JF, Genant HK (2000). Association of intramuscular cysts of the rotator cuff with tears of the rotator cuff: magnetic resonance imaging findings and clinical significance. Arthrosc J Arthrosc Relat Surg.

[12] Cullen D, Breidahl W, Janes G (2007). Diagnostic accuracy of shoulder ultrasound performed by a single operator. Australas Radiol.

